# Improving Affordability in Dermatology: Cost Savings in Mark Cuban Cost Plus Drug Company Versus GoodRx

**DOI:** 10.2196/64300

**Published:** 2024-12-13

**Authors:** Christopher G Youn, Joo Yeon Kim, Vivian B Yang, Gordon H Bae

**Affiliations:** 1 Department of Dermatology, Stanford University School of Dermatology, 450 Broadway, Pavilion C, 2nd Floor, Redwood City, CA, 94063, United States, 1 650-721-7190; 2 School of Medicine, University of California, San Francisco School of Medicine, San Francisco, CA, United States

**Keywords:** dermatology, cost, affordability, drug company, United States, US, financial burden, prescription, medication, pharmaceutical, dermatologic, burden, financial distress, health outcomes, pharmacist, pharmacy, convenience

## Abstract

This observational cost analysis was conducted to assess the efficacy of the Mark Cuban Cost Plus Drug Company (CostPlus) relative to GoodRx and found that CostPlus has significant potential to improve the financial burden of prescription medications within dermatology.

## Introduction

Prescription medication affordability is a significant issue within the United States and has been associated with medication nonadherence and negative health outcomes [1-4]. These effects are compounded in vulnerable populations, including ethnic minority groups and uninsured individuals [1]. In 2022, entrepreneur Mark Cuban launched the Mark Cuban Cost Plus Drug Company (CostPlus)—a public benefit corporation with a vertically integrated supply chain—to address rising pharmaceutical costs [2,5]. CostPlus offers medications for a 15% markup and a US $5 pharmacy fee, allowing for significant cost savings [5]. Studies have found that pharmacy coupon websites can lead to significant savings for patients within dermatology, with GoodRx providing some of the highest rates of saving [6]. However, while GoodRx is often used, medication costs can vary significantly depending on the pharmacy. CostPlus offers the benefit of obtaining medications conveniently through a mail-in-one system, yet its potential benefits have not been described. We conducted an observational cost-analysis to assess cost savings between GoodRx and the novel CostPlus model.

## Methods

### Study Design

A list of some of the most prescribed medications in dermatology was assembled from a study by Dobkin and Zirwas [6]. Prices from the CostPlus website [7]—inclusive of the pharmacy fee—were extracted on November 26, 2023. Prices on the GoodRx mobile app were extracted on November 26, 2023, from the New York, Los Angeles, and Chicago regions, as these represent the largest US metropolitan statistical areas. Reported retail prices for GoodRx were collected and averaged. Additional analyses were conducted, including a US $2.60 or US $5 fee to account for shipping costs. Wilcoxon signed rank exact tests were used, and analyses were conducted on RStudio V1.4.1106 (Posit PBC).

### Ethical Considerations

This study does not constitute human subject research and does not require institutional review board approval.

## Results

The average medication cost, excluding shipping, was US $17 (SE US $3.22) through CostPlus and US $31.21 (SE US $6.42) through GoodRx (*P*<.001; Table 1). The average medication cost using the cheapest price available from GoodRx was US $21.34 (SE US $4.71), which remained significantly different from CostPlus (*P*=.03). When a US $2.60 shipping fee per medication was included, the average cost increased to US $19.60 (SE US $3.22), remaining significantly different from the average GoodRx cost (*P<*.001) [8]. When a US $5 shipping fee, which assumed that medications were shipped individually, was included on the CostPlus platform, the average cost rose to US $22 (SE US $3.22) but remained significantly different (*P*=.007) from the average GoodRx cost. On average, the retail cost of medications was US $147.23 (SE US $36.54). CostPlus resulted in average savings of US $130.23 (SE US $33.78; 81%), while GoodRx resulted in average savings of US $116.01 (SE US $30.73; 70%; Table 2). The two lowest-priced pharmacies with GoodRx were ShopRite and Acme in New York, Vons and Ralphs in Los Angeles, and Jewel-Osco and Meijer in Chicago (Multimedia Appendix 1).

**Table 1.** Medication prices from Mark Cuban Cost Plus Drug Company (CostPlus) and GoodRx among 3 large metropolitan areas (November 26, 2023).

Drug	CostPlus drugs cost, US $	CostPlus drugs cost with US $2.60 shipping fee^a^, US $	CostPlus drugs cost with US $5 shipping fee^b^, US $	GoodRx costs, US $			
				New York	Los Angeles	Chicago	Average (SE)
Cephalexin 500 mg, 30 capsules	7.10	9.70	12.10	13.54	14.63	14.67	14.28 (0.37)
Clindamycin 1%/benzoyl peroxide 5% gel, 50-g jar	34.21	36.81	39.21	61.56	65.23	72.12	66.30 (3.10)
Clobetasol 0.05% ointment, 30-g tube	7.90	10.50	12.90	24.14	31.54	31.14	28.94 (2.40)
Doxepin 10 mg, 30 capsules	7.40	10	12.40	8.85	9.39	9.41	9.21 (0.18)
Doxycycline monohydrate 100 mg, 60 capsules	15.20	17.80	20.20	22.14	23.16	22.14	22.48 (0.34)
Doxycycline hyclate 100 mg, 60 capsules	15.80	18.40	20.80	29.76	35.34	33.95	33.02 (1.68)
Hydroxyzine 25 mg, 30 tablets	7.10	9.70	12.10	9.54	11.21	11.29	10.68 (0.57)
Imiquimod 5% cream, 30 packets	27.50	30.10	32.50	46.53	41.69	51.71	46.64 (2.89)
Ketoconazole 2% cream, 30-g tube	9.44	12.04	14.44	18.75	21.25	19.47	19.83 (0.74)
Methotrexate 2.5 mg, 30 tablets	8	10.60	13	18.25	19.04	18.04	18.44 (0.30)
Metronidazole 0.75% cream, 45 g	26.41	29.01	31.41	36.121	49.26	36.68	40.69 (4.29)
Minocycline 100 mg, 60 capsules	22.40	25	27.40	33.30	34.84	35.49	34.54 (0.65)
Prednisone 10 mg, 60 tablets	8.60	11.20	13.60	9.97	9.23	9.36	9.52 (0.23)
Tacrolimus 0.1% ointment, 60-g tube	48.65	51.25	53.65	93.02	102.03	104.93	99.99 (3.59)
Triamcinolone 0.1% ointment, 80-g tube	9.25	11.85	14.25	13.02	13.77	14.16	13.65 (0.33)

a The US $2.60 value was derived by combining a 2023 study by Patil et al [8], which identified an average of 2.7 medications per prescription, and the shipping cost of US $7 for 3 or 4 medications via CostPlus drugs.

b The US $5 shipping cost assumed that medications were shipped individually.

**Table 2.** Absolute and percentage savings through Mark Cuban Cost Plus Drug Company (CostPlus) and GoodRx (November 26, 2023).

Drug	Retail cost (US $), average (SE)	GoodRx savings (US $), average (SE; % savings)^a^	CostPlus savings, US $ (% savings)
Cephalexin 500 mg, 30 capsules	27.54 (3.17)	13.26 (0.12; 48)	20.44 (74)
Clindamycin 1%/benzoyl peroxide 5% gel, 50-g jar	319.83 (15.59)	253.52 (2.14; 79)	285.62 (89)
Clobetasol 0.05% ointment, 30-g tube	197.43 (0.26)	168.50 (0.81; 85)	189.53 (96)
Doxepin 10 mg, 30 capsules	27.70 (5.97)	18.48 (0.06; 67)	20.30 (73)
Doxycycline monohydrate 100 mg, 60 capsules	103.74 (8.27)	81.26 (0.30; 78)	88.54 (85)
Doxycycline hyclate 100 mg, 60 capsules	192.91 (17.31)	159.90 (0.68; 83)	177.11 (92)
Hydroxyzine 25 mg, 30 tablets	23.11 (1.93)	12.43 (0.19; 54)	16.01 (69)
Imiquimod 5% cream, 30 packets	387.42 (39.80)	340.78 (2.89; 88)	359.92 (93)
Ketoconazole 2% cream, 30-g tube	83.35 (8.18)	63.52 (0.55; 76)	73.91 (89)
Methotrexate 2.5 mg, 30 tablets	84.26 (5)	65.82 (0.29; 78)	76.26 (91)
Metronidazole 0.75% cream, 45 g	158.51 (15.66)	117.82 (3.71; 74)	132.10 (83)
Minocycline 100 mg, 60 capsules	91.57 (7)	57.02 (0.29; 62)	69.17 (76)
Prednisone 10 mg, 60 tablets	27.39 (1.83)	17.87 (0.08; 65)	18.79 (69)
Tacrolimus 0.1% ointment, 60-g tube	464.22 (11.70)	364.23 (1.43; 78)	415.57 (90)
Triamcinolone 0.1% ointment, 80-g tube	19.44 (1.93)	5.79 (0.15; 30)	10.19 (52)
Average	147.23 (36.54)	116.01 (30.73; 70)	130.23 (81)

a Obtained by averaging reported GoodRx retail costs in all 3 metropolitan regions.

## Discussion

We demonstrate that while use of either GoodRx or CostPlus allows for significant savings, CostPlus offers significantly higher rates of saving, even when isolating the lowest prices from GoodRx. Thus, CostPlus is a viable tool to reduce prescription medication burden within dermatology and especially among uninsured individuals or those with high-deductible health plans [5].

CostPlus may offer patients and clinicians more convenience in addition to greater cost savings. For example, the lowest prices in urban regions were derived from dozens of different pharmacies with variable prices. However, in rural areas, patients may have less pharmacy options, leading to higher costs. Furthermore, to obtain the greatest amount of cost savings for multiple prescriptions via GoodRx, patients may need to travel to various pharmacies. Transportation-related barriers can lead to delayed medical care, particularly in low-income and ethnic minority groups [9]. By shipping all prescriptions at once directly to patients, CostPlus may improve vulnerable patient populations’ access to care.

This study’s limitations include its observational nature, along with measurement of drug prices at a single time point. Additionally, a study by Patil et al [8] in 2023 found that 8% of dermatology patients are prescribed ≥5 medications, and our study did not fully assess the impact of ordering multiple medications at once. Further, our study did not assess prices from compounding pharmacies, which often offer discounted prices. Our study is also not generalizable, given that CostPlus is the first of its kind in the United States. Finally, certain common medications used in dermatology, including tretinoin and calcipotriene, are not available on CostPlus, which may limit its applicability.

## Supplementary material

10.2196/64300 Multimedia Appendix 1 Lowest-cost pharmacy with GoodRx coupons in 3 large metropolitan regions (November 26, 2023).

## Abbreviations

CostPlus: Mark Cuban Cost Plus Drug Company
