# Supplementary material for: Improving Affordability in Dermatology: Cost Savings in Mark Cuban Cost Plus Drug Company Versus GoodRx
Source: JMIR Dermatol. 2024 Dec 13;7:e64300. doi: 10.2196/64300 (PMC11661688; doi:10.2196/64300)
Supplement: Multimedia Appendix 1 [file derma-v7-e64300-s001.docx]

| **Drug** | **New York** | **Los Angeles** | **Chicago** |
| --- | --- | --- | --- |
| Cephalexin 500 mg, 30 capsules | Wegman’s | Costco | Costco |
| Clindamycin 1%/benzoyl peroxide 5% gel, 50-g jar | ShopRite | Walmart | Walmart |
| Clobetasol 0.05% ointment, 30-g tube | ShopRite | Vons | Meijer |
| Doxepin 10 mg, 30 capsules | Acme | Vons | Jewel-Osco |
| Doxycycline monohydrate 100 mg, 60 capsules | Acme | Ralphs | Jewel-Osco |
| Doxycycline hyclate 100 mg, 60 capsules | Wegman’s | Vons | Meijer |
| Hydroxyzine 25 mg, 30 tablets | Walmart | Rite Aid | Meijer |
| Imiquimod 5% cream, 30 packets | Wegman’s | Ralphs | Walgreens |
| Ketoconazole 2% cream, 30-g tube | ShopRite | Vons | Meijer |
| Methotrexate 2.5 mg, 30 tablets | Acme | CVS | Jewel-Osco |
| Metronidazole 0.75% cream, 45 g | ShopRite | Ralphs | Marianos |
| Minocycline 100 mg, 60 capsules | Acme | Vons | Jewel-Osco |
| Prednisone 10 mg, 60 tablets | CVS | CVS | CVS |
| Tacrolimus 0.1% ointment, 60-g tube | ShopRite | Ralphs | Marianos |
| Triamcinolone 0.1% ointment, 80-g tube | Stop&Shop | CVS | CVS |

Table S1

Lowest Cost Pharmacy with GoodRx Coupons in Three Large Metropolitan Regions, November 26, 2023

*One-time promotions offered by GoodRx were excluded as were duplicate pharmacies under the same parent company (e.g Albertsons, Vons, and Pavilions are all owned by Albertsons)
